# Plasma-Enhanced Graphene Coatings on Ti-6Al-4V: Insights from Non-Destructive Characterization

**DOI:** 10.3390/ma19040774

**Published:** 2026-02-16

**Authors:** Victor J. Sagrero, Fnu Gorky, Vashanti Storr, Fernando M. de Oliveira, Héctor G. Carreón, María L. Carreón

**Affiliations:** 1 Instituto de Investigación en Metalurgia y Materiales, Universidad Michoacana de San Nicolás de Hidalgo, Morelia 58030, Michoacán, Mexico; victor.sagrero@umich.mx (V.J.S.); hcarreon@umich.mx (H.G.C.); 2 Ralph E. Martin Department of Chemical Engineering, University of Arkansas, Fayetteville, AR 72701, USA; gorky@uark.edu (F.G.); vstorr@uark.edu (V.S.); 3 Institute for Nanoscience and Engineering, University of Arkansas, Fayetteville, AR 72701, USA; fmaiade@uark.edu

**Keywords:** plasma-enhanced CVD (PECVD), micron-sized graphene coatings, thermoelectric power, Ti-6Al-4V ELI grade

## Abstract

In this work, the deposition of graphene coatings on substrates of an ELI grade Ti-6Al-4V alloy was carried out using the Plasma Enhanced Chemical Vapor Deposition (PECVD) technique. The purpose of this study was to improve the surface properties of the material. The characterization of the material was carried out by non-destructive techniques, such as Raman Spectroscopy and Thermoelectric Potential. A preliminary characterization of Ti substrates was carried out by Raman spectroscopy. Conversely, thermoelectric potential tests were conducted using three distinct tip systems and four different temperature gradients. Lastly, some surface roughness measurements were conducted on all samples, both coated and uncoated. Graphene micro-structured coatings were obtained using a plasma-activated mixture of hydrogen and methane gases with an equimolar feed ratio (1:1 H_2_:CH_4_) at a temperature of 850 °C and a plasma exposure of 150 Watts and duration of 15 min. Raman spectra verified the presence of uniform micrometric graphene on the surface of Ti substrates. Graphene-coated Ti-6Al-4V ELI substrates exhibited Seebeck coefficient values indicating metallic-like behavior and suitability for thermoelectric sensing. In the eddy current analyses, it was found that low frequencies provided the highest sensitivity for differentiating between samples. An inverse relationship was identified between substrate thickness and phase angle, and a direct relationship with calculated electrical conductivity was also identified. This direct relation is attributed to penetration depth and interactions due to the chemical nature of the substrate and coating. Despite a slight increase in surface roughness after graphene deposition, values remained comparable to the base alloy, preserving compatibility for biomedical integration. Thermoelectric potential measurements revealed enhanced sensitivity to surface morphology and interfacial effects when high-sensitivity probe configurations were employed. These results support potential applications in implantable or wearable temperature sensors, energy harvesting devices, and smart biomedical interfaces. The thickness of the graphene coating was also characterized by SEM, which showed that the films deposited by PECVD are about 1 micron thick.

## 1. Introduction

Titanium alloys are frequently utilized as biomaterials due to their mechanical properties and exceptional corrosion resistance [1,2].

Ti-6Al-4V is the most prevalent titanium alloy. The alloy’s combination of α and β phases endows it with fatigue resistance and ductility. The ELI grade of this alloy is defined as “extra low interstitials,” which refers to the presence of interstitial atoms, such as Fe, O, N, and H, within its lattice. This composition enhances the alloy’s fracture strength [3,4,5].

Several authors [6,7,8] have studied the effects of various mechanical and chemical factors on the surface of this alloy to improve its performance as a biomaterial. These factors include corrosion resistance and improved morphology to promote cell growth and adhesion to bones and other tissues.

However, the wear and corrosion resistance of the Ti-6Al-4V ELI alloy as a biomaterial are still under study, mainly because its practical use involves constant stress and continuous exposure to body fluids rich in ions that induce corrosion.

To address these limitations, the use of coatings has emerged as a solution to the aforementioned drawbacks. These coatings are designed to protect the substrate from various phenomena, including mechanical wear, oxidation, and corrosion, among others [9,10,11].

A variety of techniques have been developed for the purpose of generating these coatings. The selection of the technique to employ is contingent upon the type of substrate (i.e., polymers, ceramics, metal alloys, or composite materials). Similarly, each technique possesses its own set of advantages and limitations in terms of the process that must be followed to synthesize and deposit the coating material.

Among these approaches, the chemical vapor deposition (CVD) technique is widely used and well established. It is comprised of a precursor gas that flows into a closed chamber. In this environment, the substrate is heated, and the deposition of the coatings occurs as a result of the pyrolysis reaction of the precursor in the gas phase. Plasma-enhanced chemical vapor deposition (PECVD), while sharing the similarity of feeding the precursors in the gas phase, differs considerably due to the use of an electric source required to create a rich chemistry environment by the collision of neutrals with electrons. This rich chemical environment contains electrons, ions, radicals and vibrational excited species. Furthermore, a pressure reduction system is necessary to stably maintain a homogenized plasma reactive environment [12,13,14]. PECVD is a process that facilitates the deposition of a diverse array of chemical species that are hard to dissociate. Moreover, PECVD offers the advantage to occur at lower temperatures and faster deposition times compared with conventional thermal methods [15,16].

This process does not involve the use of costly or sophisticated precursors, making it a cost-effective solution [17,18]. Among the coatings applied to metallic substrates, graphene has emerged as a particularly salient option due to its unique physical and chemical properties, as well as its ease of synthesis.

Thin-film graphene deposition onto a variety of substrates has been in the limelight in recent years due to its robustness and outstanding properties. For instance, by leveraging graphene’s excellent electrical conductivity and mechanical flexibility, the research community has demonstrated implantable and wearable temperature sensors capable of monitoring physiological parameters with high sensitivity and rapid response times [19,20]. Similarly, graphene-coated electrodes and membranes have been merged into energy harvesting systems such as piezoelectric generators to efficiently convert thermal variations into usable electrical power [21,22]. In the biomedical sector, these same deposition techniques have given rise to smart interfaces that impeccably integrate with tissue, enabling real-time tracking of biochemical signals [23,24,25,26]. Beyond sensing and harvesting, thin graphene films have also been explored to enhance the performance and longevity of energy-storage devices: Their high surface area and conductivity improve charge-discharge rates in batteries and supercapacitors [27,28,29]. Finally, when applied as ultra-thin protective coatings, graphene offers exceptional resistance to corrosion, protecting metal surfaces in harsh environments without sacrificing weight or thickness [30].

It has been demonstrated that graphene exhibits high thermal (above 3000 W/m·K) and electron mobility (2.5 × 10^5^ cm^2^/V·s), as well as superb mechanical strength (Young’s modulus of 1 TPa and intrinsic strength of 130 GPa). This coating is also a highly suitable option for biomedical materials, such as the ELI grade Ti-6Al-4V alloy, due to its inherent properties, including chemical resistance, thermal stability, gas impermeability, and antibacterial potential [31,32,33,34,35].

Different authors, such as Wang et al. [36], have reported on the use of graphene as a coating for biomaterials. They used PECVD to deposit graphene on Ti-6Al-4V substrates and found that the behavior of different bacterial and fungal pathogens, such as *P. gingivalis* and *C. albicans*, changed when they came into contact with the alloy coated with graphene on its surface. These pathogens tended to deform and even shrink, a behavior which was not observed in the bare material by itself.

Romo-Rico et al. [37] also used PECVD to deposit graphene on a CoCr alloy, which is used as a biomaterial. They found that, in addition to promoting cell adhesion, this type of coating had an antibacterial effect against Staphylococcus aureus and Pseudomonas aeruginosa.

Similarly, Malhotra et al. [38] evaluated the corrosion resistance of a Ti-6Al-4V ELI system with nanostructured graphene coatings and found that the corrosion rate decreased by up to five times compared to the alloy without coatings in a 0.5 M NaCl environment with 2 ppm fluoride.

This technique has been studied for the growth of coatings of different chemical species on Ti-6Al-4V substrates to improve properties such as corrosion resistance. Such is the case of Oliveira et al. [39], who used plasma-assisted CVD to coat this alloy with nitride films. They achieved higher impedance values with these different nitride systems than with the base material.

Similarly, Cho et al. [40] used PECVD to deposit amorphous carbon coatings on Ti-6Al-4V. These coatings allowed for corrosion rates 30 times lower than the bare alloy and exhibited improved biocompatibility during in vitro tests with cell cultures.

In terms of characterization, Raman spectroscopy has emerged as a pivotal technique for the analysis of carbon-based materials, including graphene.

This technique is considered as a rapid, non-destructive, and high-resolution way to characterize graphene. It is based on the effect of inelastic light scattering, otherwise known as “Raman scattering”. This phenomenon occurs when light interacts with the molecules of a material and scatters at different energy levels corresponding to the vibrational modes of the molecule [41,42].

Ferrari & Basko [43] detail the information that can be obtained from Raman spectra of graphene. They highlight the usefulness of the technique since it can determine the number and orientation of synthesized graphene layers, their quality, and the types of their edges. Most importantly, it provides knowledge about sp^2^ carbon allotropes since they are the fundamental unit of graphene. Thus, these authors provide extensive information about the technique’s usefulness in determining graphene’s structure and chemical properties, as well as a scientifically rigorous approach to interpret results and the valuable information obtained from them, also highlighting the importance and usefulness of the Raman spectroscopy technique for the study of graphene.

Eckmann et al. [44] used Raman spectroscopy to analyze defects and disorder in graphene by examining the intensities of peaks in this material’s characteristic bands and the relationships between them. They associate the ratio of intensities in the D and D’ bands with defects related to vacancies and boundaries. For vacancies and boundaries, the presence of these defects is indicated by a decrease and a minimum in the ratio of intensities, respectively.

The non-destructive thermoelectric power technique (TEP) is predicated through the Seebeck effect, which establishes that if a thermal gradient is induced between a dissimilar junction, a potential voltage will be produced in the metallic sample that is integrated into such a dissimilar junction. This technique employs the thermoelectric potential of a material to characterize its conductive properties, as any defects or perturbations within the crystal lattice, such as elements in solid solution, precipitates, or dislocations, can affect this potential. These defects contribute to changes in the material’s electrical and elastic properties, thereby inducing a variation in the thermoelectric potential [45,46].

Park et al. [47] reported using this non-destructive technique to evaluate the content of interstitial elements in steels, such as AISI 1090, as well as to analyze residual stresses in aluminum, nickel, and bronze alloys. In both cases, they report linear correlations with respect to the evaluation variables: % nitrogen for evaluating interstitial elements and stress values for analyzing residual stresses.

Carreón et al. [47,48] used the thermoelectric potential technique to analyze wear in Ti-6Al-4V alloys and evaluate aging and precipitation formation. This demonstrated the sensitivity of the technique to surface analysis of this class of materials.

Eddy currents, also known as Foucault currents, are a non-destructive inspection technique that uses a magnetic field generated by an electric current in a coil to detect surface and subsurface discontinuities.

The issue of conductivity changes in alloys, such as nickel-based superalloys and Ti-6Al-4V, continues to be studied. It appears that conductivity changes due to subtle microstructural changes, such as the transition from long-range to short-range order or changes in the numerical density and size of precipitates [49].

Eddy currents testing (ECT) involves the flow of an electric current through a circuit that operates the primary coil. However, all electric currents encounter resistance to their flow. In an alternating current system, this resistance is represented by impedance (Z), which is expressed as the sum of a real part (R) and an imaginary part (X). The real part is the common resistance, and the imaginary part is called reactance. There are two types of reactance, inductive (L) and capacitive (C), due to the presence of inductors and capacitors, respectively [50].

Rosen and Horowitz [51] identified a decrease in the conductivity of 2024 aluminum alloys during the alloys’ aging process at temperatures between 21 and 190 °C. This decrease is due to the formation of GP and GPB zones, also called Guinier–Preston and Guinier–Preston–Bagaryatsky zones. These zones form rapidly during tempering and are predominantly composed of solute elements. Essentially, they are tiny agglomerations of atoms that precipitate in the matrix during the initial stages of precipitation hardening. The kinetics of forming these zones are governed by the mobility of copper and magnesium atoms, as well as their interaction with vacancies. The formation of these solute atom agglomerations directly influences conductivity measurements in eddy current tests.

Surface roughness depends heavily on the manufacturing method used. This property enables us to understand how an object interacts with its environment, as it is directly related to phenomena such as friction, wear, and the adhesion of bodies to each other [52,53].

In the specific context of using Ti-6Al-4V ELI as a biomaterial, surface roughness is important because cells grow preferentially on rough surfaces. This affects the rate and quality of new tissue formation (Deligiani et al. [54]).

Although graphene coatings deposited by PECVD on titanium alloys have been reported, most studies focus primarily on antibacterial performance or corrosion resistance. Less attention has been given to the functional electrical response of graphene-coated Ti-6Al-4V ELI, particularly using non-destructive techniques. In addition, Seebeck coefficient values for PECVD-grown nanographene on this alloy remain limited. A better understanding of these thermoelectric properties is important for emerging biomedical applications, where coated titanium surfaces may serve not only as protective layers but also as sensing or energy-responsive interfaces. In this work, we aim to optimize deposition parameters for graphene growth on Ti-6Al-4V ELI substrates by PECVD and to evaluate the structural and functional properties of the resulting coatings using Raman spectroscopy, thermoelectric potential measurements, eddy current analysis, and surface roughness characterization.

## 2. Materials and Methods

### 2.1. Materials

This investigation was carried out using three sets of samples of Ti-6Al-4V alloy ELI grade. Each set contained three samples with different thicknesses (1.6 mm, 3.2 mm, and 7 mm).

Furthermore, titanium substrates with a diameter of 15 mm and a thickness of 0.125 mm were employed in the preliminary tests to ascertain the optimal parameters for graphene growth by PECVD.

### 2.2. CVD and PECVD Preliminary Tests and Deposition on Ti-6Al-4V ELI Grade Samples

The samples were mirror polished with sandpaper meshes of 240 and 600; they were sonicated in an acetone solution for 10 min to remove surface contamination. The samples were then oven dried for 4 h at 80 °C before being placed inside the reactor chamber, which was subsequently pumped to vacuum.

**Figure 1:**
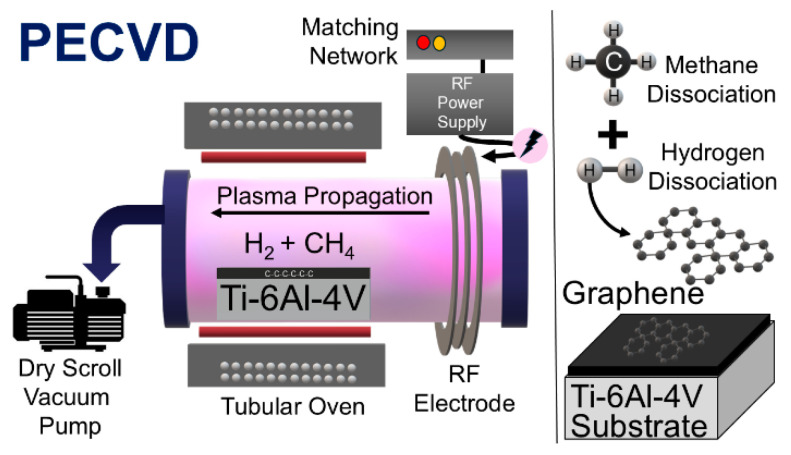
In-house-built radio frequency plasma reactor with a sample inside.

The experiments were conducted in an in-house-built plasma reactor (Figure 1) [55], maintaining a constant reaction pressure of 0.1 torr. For the preliminary study, we conducted CVD and PECVD on small titanium substrates (15 mm diameter, 0.125 mm thickness). The Ti substrates were heated in a tubular furnace at temperatures ranging from 425 to 850 °C, with a ramp rate of 10 °C min^−1^. A flow of 20 sccm was controlled via mass flow controller (MFC) with an equimolar mixture of methane and hydrogen. The samples were maintained at 850 °C for 2 h, followed by plasma exposure between 15 and 30 min, respectively, at 150 W plasma power. The samples were then cooled to room temperature at a rate of 20 °C min^−1^. Based on the preliminary study results, the optimal parameters for graphene synthesis were applied to various sizes of Ti-6Al-4V with various thickness of 1.6 mm, 3.2 mm, and 7 mm.

### 2.3. Raman Characterization of Preliminary Ti Samples

The graphene films were characterized using a Horiba Jobin-Yvon LabRAM HR Raman spectrometer (Lille, France). The system was equipped with an Olympus microscope and lenses and used a 632 nm laser with a collection time of 10 s at room temperature for the measurement.

### 2.4. TEP Characterization of Ti-6Al-4V ELI Grade Samples

**Figure 2:**
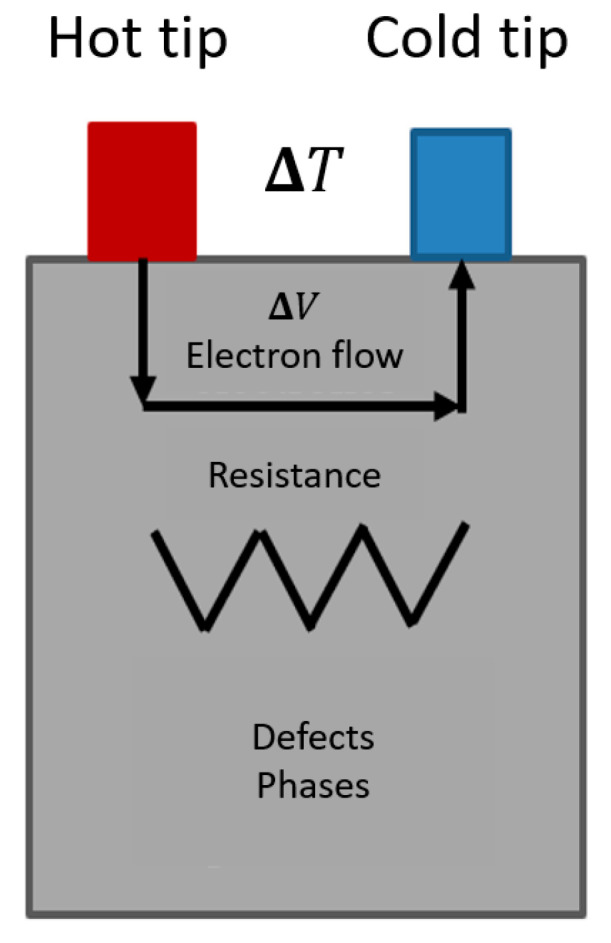
Schematic of the hot tip TEP technique.

The thermoelectric power measurements of the ELI grade Ti-6Al-4V samples, with the graphene coating already deposited, were obtained by employing a Thermo-Sorter apparatus manufactured by Walker Scientific, Inc. (Worcester, MA, USA). This equipment uses the hot tip technique (Figure 2), which involves the use of three different pairs of tips. The pair of tips employed were Cu-Cu, Cu-Ni and Cu-Au.

In addition to the three aforementioned systems, it is important to acknowledge that the measurements were obtained under varying thermal gradients (∆T = 50 °C, 40 °C, 30 °C, and 20 °C) for each pair of tips utilized. This procedure was repeated for all nine studied samples, with a total of 30 measurements being recorded for each sample.

It should be noted that the recorded measurements provide only µV values, that is to say, only voltage variations. To obtain the absolute value, also referred to as the Seebeck coefficient [45], it is necessary to refer to its formula described in Equation (1) [45].

S = ∆V/∆T(1)

The presented equation elucidates the phenomenon known as the Seebeck effect, which dictates that the generation of a thermal gradient within a system of differing composition will yield a voltage difference that can be quantitatively measured by means of a voltimeter [45]. Given that the measurements indicate the value of the voltage difference, it is important to determine the thermal gradient utilized to obtain the absolute potential. This is achieved by dividing the measured voltage value by the temperature difference, as the previous equation shows, thereby yielding the value of the Seebeck coefficient (S).

### 2.5. Conductivity Measurements Using Eddy Currents Testing (ECT)

**Figure 3:**
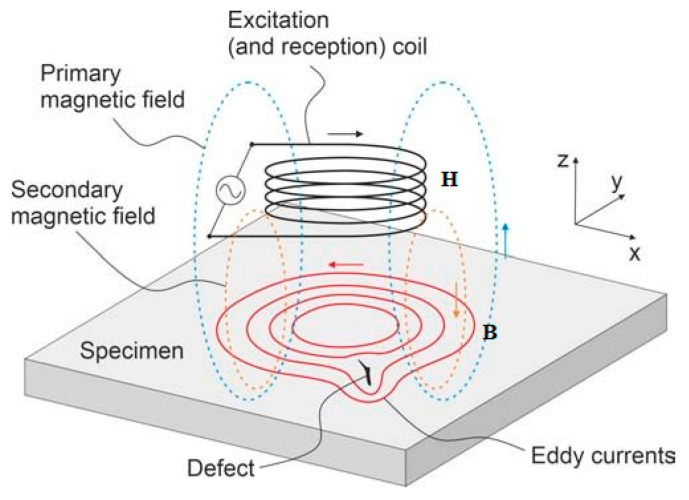
A schematic diagram of detection at material imperfections by electromagnetic sensing. The excitation coil generates a primary magnetic field (blue dashed lines) that induces eddy currents in the conductive specimen (red lines). These currents produce a secondary magnetic field, which is distorted in the presence of a defect. Arrows indicate field and current directions, and the x–y–z axes define the spatial orientation. H is the magnetic field strength and B represents magnetic flux density.

The OLYMPUS NORTEC 500D (Olympus NDT Inc., Houston, TX, USA) was used to characterize Ti-6Al-4V grade ELI samples with graphene on their surface using the eddy current technique. Measurements were taken at various inspection frequencies across all parts, spanning from 5 kHz to 6 MHz with pencil-type probes. As demonstrated in Figure 3, the operating principle of the measuring tubes for the eddy current technique with the test specimen is illustrated.

In addition to directly obtaining the phase angles with the aforementioned equipment, these values were used to indirectly obtain electrical conductivity data by associating the phase angles of paramagnetic materials, such as Ti-6Al-4V, with known conductivity. The materials used were the following copper alloys: CDA 110 (101% IACS) and CDA 360 (26% IACS); the aluminum alloy Al5086 (31% IACS); the stainless steel SS304 (2.5% IACS); and the commercial Ti-6Al-4V alloy (1% IACS).

Finally, we calculated the penetration depth of the induced currents for both the substrate (Ti-6Al-4V ELI) and the coating (graphene) using Equation (2) [50], which is presented below.

(2) $$\delta =\frac{1}{\sqrt{\pi f\mu \sigma }}$$

where said depth is calculated in meters, f represents the inspection frequency in Hz, µ represents the magnetic permeability of the material in H/m, and σ represents the electrical conductivity in S/m.

The magnetic permeability values are 0.000001256 H/m for both the titanium alloy and graphene, while the electrical conductivity values are 561.79775 S/m and 10,000 S/m, respectively.

The same equation was used for both materials because they are both non-ferromagnetic conductors. This is reflected by the use of the same magnetic permeability value, which indicates that this property is common to both materials.

At 50 kHz, the calculated penetration depth for Ti-6Al-4V is on the order of several millimeters, which is comparable to or greater than the substrate thickness range investigated (1.6–7 mm) and several orders of magnitude larger than the nanographene coating thickness (~1–2 µm). This confirms that the eddy-current response is dominated by the bulk substrate while remaining sensitive to near-surface conductivity modifications.

### 2.6. Surface Roughness Measurements

Surface roughness measurements of the samples were conducted using a Mitutoyo SJ-210 roughness tester, manufactured by Mitutoyo Corporation (Aurora, IL, USA). Using this instrument, a scan was performed with 10 measurements across the surface of the samples to obtain average roughness values (Ra).

### 2.7. Scanning Electron Microscopy (SEM) Characterization

For the characterization of the system with the Ti-6Al-4V alloy substrate ELI grade and the graphene coating on its surface, the samples were observed in a Jeol JSM-7600 field emission scanning electron microscope (FESEM), manufactured in Japan (Tokyo).

## 3. Results

### 3.1. Preliminary Tests of PECVD on Ti-Substrate

Based on the preliminary results obtained from the Ti-substrate, Raman spectroscopy was conducted to measure the D, G, and 2D bands on the samples. From the Raman spectroscopic interpretation, the D band is defined as a defect-activated Raman mode arising from breathing-like vibrations of sp^2^ carbon rings and this band appears when the lattice is disordered (edges, vacancies, functional groups, sp^3^ bonding and other defects) [56]. The G-band is denoted as in-plane E_2g_ stretching mode of sp^2^ bonded carbon (C-C) [57]. Finally, the 2D band represents the second-order, two-phonon Raman mode that is defect independent. Specifically, the 2D band shape, width, and intensity are highly sensitive to the number of graphene layers and their stacking order [58,59].

**Figure 4:**
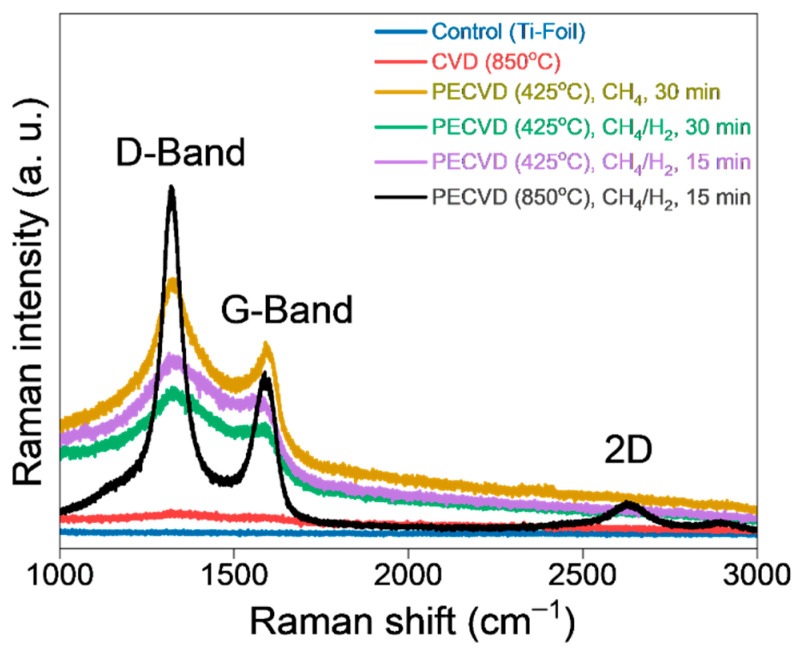
Raman spectra of preliminary samples (Ti substrates) upon exposure to varying conditions.

Based on Figure 4, the Raman spectra of these samples provided valuable insights into the characteristics of the synthesized graphene. In the control Ti-substrate and samples subjected to CVD at 850 °C with a 10 sccm flow of CH_4_, no D, G, or 2D bands were detected. This indicated the absence of graphene. Instead, the film exhibited a brown coating, revealing the presence of surface coking on the surface.

Further investigation involved varying PECVD parameters. Samples processed with PECVD at 425 °C using 10 sccm of CH_4_ for 30 min of plasma exposure, and those with 20 sccm of an equimolar feed of CH_4_/H_2_ for 30 min and 15 min of plasma exposure, respectively, showed the presence of D and G bands but lacked 2D bands. The D band observed at approximately 1350 cm^−1^ is associated with defects or disorder in the graphene lattice [37,38], while the G band—around 1580 cm^−1^—represents the in-plane vibration of sp^2^-bonded carbon atoms, indicating the crystalline quality of the graphene.

The optimal parameters for graphene synthesis were identified as PECVD at 850 °C with a 20 sccm flow of an equimolar mixture of CH_4_ and H_2_ for 15 min of plasma exposure. Under these conditions, more pronounced and sharp peaks of the D, G, and 2D bands were observed. The 2D band, occurring around 2700 cm^−1^ (see Figure 4), is a second-order overtone of the D band and is commonly used to assess the number of graphene layers and their stacking order. The presence of a broadened 2D band with wider FWHM indicated few-layer, defect-rich graphene [55,60].

Furthermore, the I_2D_/I_G_ and I_D_/I_G_ ratios were obtained, which, according to the extensive literature on Raman spectroscopy characterization of graphene, indicate the stacking of layers (monolayer or multilayer) of the material and the uniformity (number of defects in the film), respectively [32,61]. In the case of the first mentioned ratio, if its value is close to 0, it is a multilayer coating, whereas if its value increases, it indicates that the film consists of few graphene layers [31,62]. In contrast, an elevated I_D_/I_G_ ratio is indicative of a heightened prevalence of defects associated with grain boundaries within the structure [43,44]. In this work, the I_2D_/I_G_ values obtained ranged from 0.46 to 0.61. These values indicate the formation of a limited number of layers in the coating. However, there are enough layers to provide a thickness of an order of magnitude of microns. For the I_D_/I_G_ ratio, the values ranged from 1.18 to 1.32. The values obtained from five measurements can be found in Table S1 of the Supplementary Materials. This suggests that the films obtained exhibit slight amounts of defects related to grain boundaries [63,64,65], thereby indicating a uniform distribution of graphene deposited on titanium substrates. The values obtained for the D, G, and 2D band intensities were collected at five distinct positions of the same sample. It should be noted that this sample was previously identified as exhibiting optimal parameters, as referenced in the preceding paragraph. To further elucidate the effect of growth time under optimized parameters, additional experiments were performed and are discussed in the next sections.

### 3.2. Growth Time Influence in Optimal PECVD Parameters

**Figure 5:**
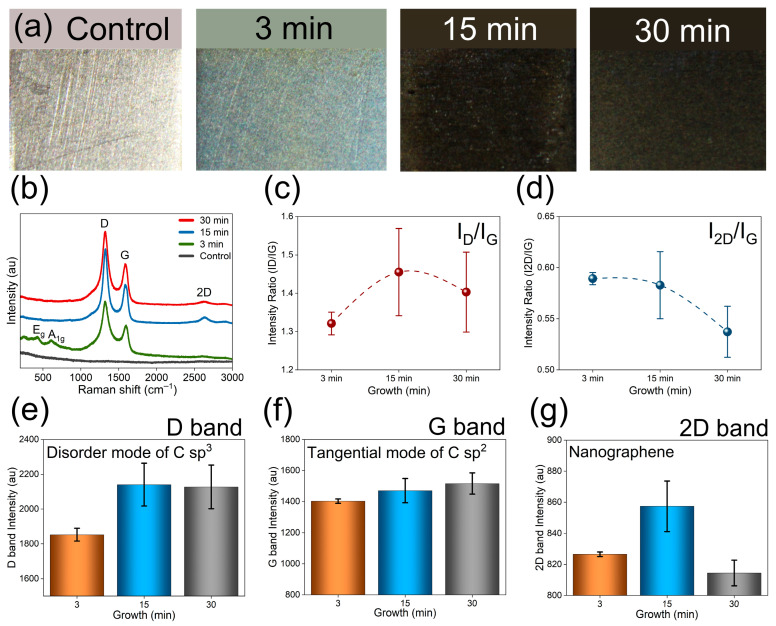
Comparative analysis of growth (min); (**a**) PECVD deposition on control (Titania) sample with various time (3–30 min); (**b**) Raman spectroscopy of PECVD samples; (**c**) intensity ratio (I_D_/I_G_); (**d**) intensity ratio (I_2D_/I_G_); (**e**) bar plot on D band; (**f**) bar plot on G band; (**g**) bar plot on 2D band intensity.

Building on our preliminary observation that 2D bands appeared after 15 min of plasma exposure (at 850 °C), we conducted additional experiments at 3, 15, and 30 min on Ti-6Al-4V substrates and compared both their visual appearance and Raman spectra (Figure 5). Visual inspection showed evident time-dependent changes: The bare substrate appeared grey-silver, after 3 min the sample showed two distinct color regions (grey-blue at the edges and orange-reddish towards the direction of plasma), after 15 min the surface was brighter black, and after 30 min the film had a matte black finish (Figure 5a). Raman analysis revealed that the 2D band was most prominent for the 15- and 30-min samples, with 15 min giving the optimal signal. The 3 min film showed two additional bands attributable to rutile titania (E_g_ at 447 cm^−1^ and A_1g_ at 612 cm^−1^), indicating non-uniform carbon coverage with 3 min plasma exposure; these rutile features disappeared for the 15- and 30-min samples (Figure 5b). The Raman signatures overall are consistent with the growth of nanographene [61,66,67,68] (NG) which is primarily described as nanoscale, few-layer graphene fragments with an amorphous variant characterized by a high density of defects. Specifically, the spectra exhibit a strong D band (defect mode) and relatively weaker G and 2D features [44,69]; the I_D_/I_G_ ratio (~1.4) indicates substantial disorder and amorphous characteristics, which is plausibly induced by the reactive plasma environment (electrons, ions, radicals, neutrals, photons, etc.) [56]. The I_2D_/I_G_ ratio was 0.6, consistent with few-layer graphene [70,71]. Comparing the samples, the D band (disorder, largely sp^3^ related [56]) reached its highest intensity in the 15 min film while the G band (tangential sp^2^ mode [57]) remained comparatively lower, consistent with a material that is rich in sp^2^ bonding but heavily defected rather than graphite-like stacked layers [61]. A small monolayer-related 2D feature was strongest at 15 min, weaker at 3 min, and weakest at 30 min (Figure 5e–g). Taken together, the visual and spectroscopic data indicated that 15 min of PECVD provided the best balance between uniform coverage and nanographene presence (see Figure 5a–g).

**Figure 6:**
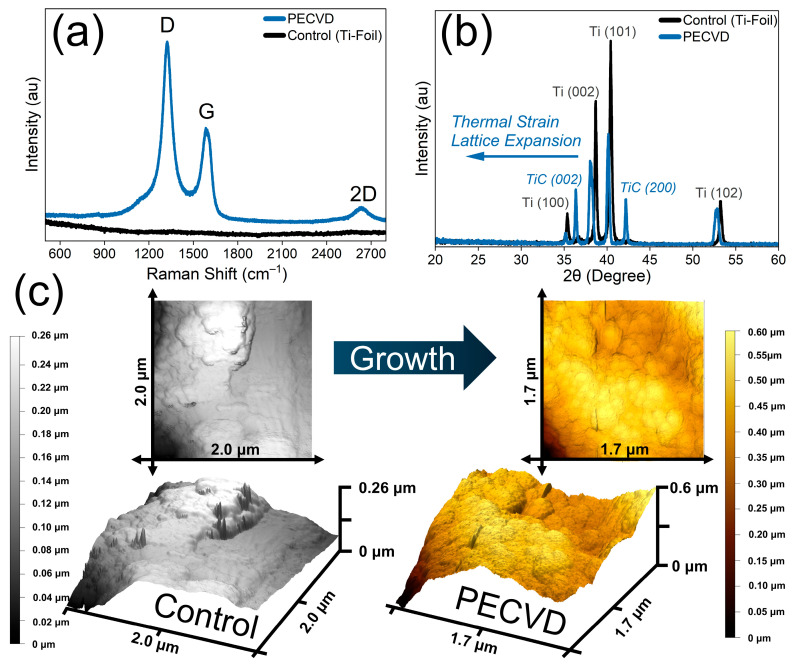
Control Vs. PECVD thin film morphological characteristics; (**a**) Raman spectroscopy; (**b**) X-ray Diffraction; (**c**) atomic force microscopy (AFM), plane XY = topography, plane XYZ (gradient represent *Z*-axis, height in micrometer); Control = Ti Foil, PECVD = 15 min plasma exposure at 150 Watts, equimolar feed ratio (CH_4_/H_2_ = 1), oven temperature = 850 degrees Celsius, pressure = 0.18 Torr.

After determining the optimal PECVD conditions for graphene growth on Ti-6Al-4V substrates, we performed additional structural and morphological characterization. Raman spectroscopy supported the presence of a few layers of graphene on the surface (Figure 6a), indicating the presence of nanographene. X-ray diffraction comparing the control and PECVD samples showed a reduction in the intensities of the Ti-foil diffraction peaks and the emergence of two new peaks of titanium carbide (TiC (002) and TiC (200)). Those XRD peaks shifted to lower 2θ values, consistent with lattice expansion or strain resulting from heating to 850 °C and from carbon incorporation into the substrate lattice; this shift is, therefore, a meaningful indicator of structural change despite that the limited surface sensitivity of XRD as lower angle was experimentally limited (Figure 6b). We did not observe strong graphite carbon (001) reflections, only a weak feature near 19.5° 2θ in the PECVD sample, indicating that bulk XRD is less sensitive to thin surface films than Raman spectroscopy. Atomic force microscopy revealed an increase in surface height (of +0.34 µm in PECVD sample) relative to the control and showed a more amorphous morphology with fewer crystalline domains (Figure 6c). The 3D height profiles revealed a clear increase in surface height and a broader height distribution for the PECVD sample, indicating deposition and film formation on the substrate. Notably, the PECVD surface lacked well-defined terraces, step edges, suggesting the absence of long-range crystalline order. Instead, the observed morphology is consistent with nanographene growth with high defect, in agreement with Raman spectroscopy results showing a strong D band and reduced 2D intensity. Finally, these observations are consistent with the known effects of energetic plasma exposure: Ion bombardment and plasma species can introduce defects and promote amorphous carbon formation [56,72,73] which helps explain the reduced crystallinity observed in the PECVD films.

### 3.3. Thermoelectric Power (TEP) Characterization of Nanographene-Coated Ti-6Al-4V ELI Grade Samples

The thermoelectric power characterization was conducted on nine nanographene-coated Ti-6Al-4V ELI samples using three electrode pairs: Cu–Cu, Ni–Cu, and Au–Cu. For each configuration, measurements were taken at thermal gradients of 20, 30, 40, and 50 °C, ensuring a systematic analysis across different conditions.

**Figure 7:**
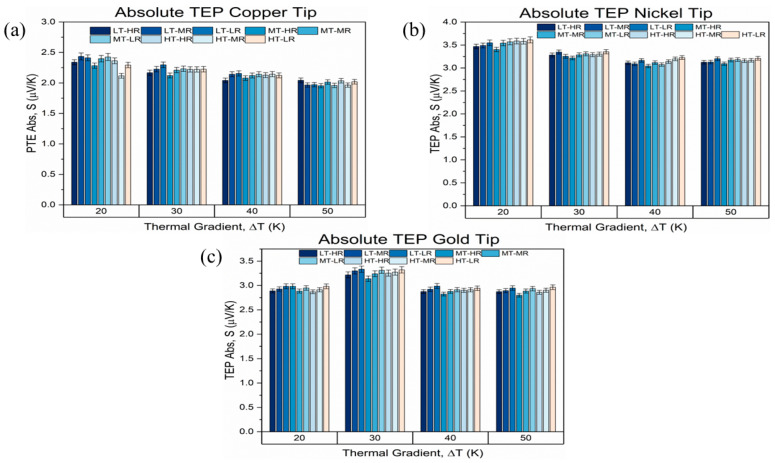
TEP values for samples with low (LT), medium (MT) and high (HT) substrate thicknesses, and low (LR), medium (MR) and high (HR) surface roughness. (**a**) Values for all samples with Cu tip. (**b**) Values for all samples with Ni tip. (**c**) Values for all samples with Au tip.

The Cu–Cu system exhibited minimal sensitivity, with negligible variation across samples and thermal gradients (Figure 7a). This suggests that copper electrodes are inadequate for detecting subtle changes in the Seebeck coefficient in this system.

Conversely, the Ni–Cu configuration showed a clear inverse relationship between thermal gradient and the measured Seebeck coefficient (Figure 7b). In this case, the values decreased as ∆T increased, revealing a higher responsiveness of this electrode pair. Furthermore, samples with medium-thickness (MT) substrates consistently presented lower Seebeck values, particularly those prepared with higher roughness (HR) finishes. This behavior contrasts with the high-thickness (HT) samples, which yielded the highest absolute values of TEP.

Figure 7 indicates that the absolute Seebeck coefficient depends on the electrode pair and the applied thermal gradient. In particular, Ni–Cu can exhibit higher average S values at low ΔT (e.g., 20 °C). However, the Au–Cu configuration displays the clearest discrimination among samples and the most reproducible dependence on ΔT and is, therefore, identified as the most sensitive electrode system in this study. These results suggest a stronger interaction between the gold-tipped system and the microstructure or defect distribution within the coating and its interface with the substrate.

In order to assess the experimental dispersion, standard deviations obtained from the 30 repeated measurements performed for each sample and electrode configuration were incorporated as error bars in Figure 7. Although the absolute variations in the Seebeck coefficient are moderate (1.9–3.4 µV/K), systematic trends become evident when using high-sensitivity electrode pairs, particularly the Au–Cu system.

While the Cu–Cu tips exhibit negligible discrimination among samples, the Au–Cu configuration reveals reproducible differences associated with both substrate thickness and surface roughness, as shown in Figure 7c. These variations are attributed to the dominant contribution of the bulk Ti-6Al-4V ELI substrate to the thermoelectric response, given that the nanographene film thickness is limited to 0.9–1.9 µm, whereas substrate thickness ranges from 1.6 to 7 mm.

Statistical validation of these trends was performed using two-factor ANOVA and correlation analysis (Tables S4–S6, Supplementary Materials), confirming surface roughness exerts a measurable, although moderate, influence on the Seebeck coefficient.

Across all electrode systems, consistent patterns were observed: Seebeck coefficient values ranged between 1.9 and 3.4 µV/K, indicating metallic-like behavior.

The Raman-derived intensity ratios provide important insight into the structural origin of the measured thermoelectric response. The high I_D_/I_G_ values (1.18–1.32) indicate a defect-rich nanographene coating with a high density of grain boundaries, vacancies, and plasma-induced disorder, while the I_2D_/I_G_ ratios (0.46–0.61) are consistent with few-layer nanographene rather than bulk graphite. Such defect-induced disorder enhances electron scattering within the coating, which limits carrier mobility and prevents the nanographene layer from dominating the thermoelectric response. As a result, the measured Seebeck coefficients (1.9–3.4 µV/K) remain metallic-like and primarily governed by the Ti-6Al-4V substrate, with the nanographene acting as a surface-modulating layer rather than as an independent thermoelectric material.

### 3.4. Eddy Currents Characterization

**Figure 8:**
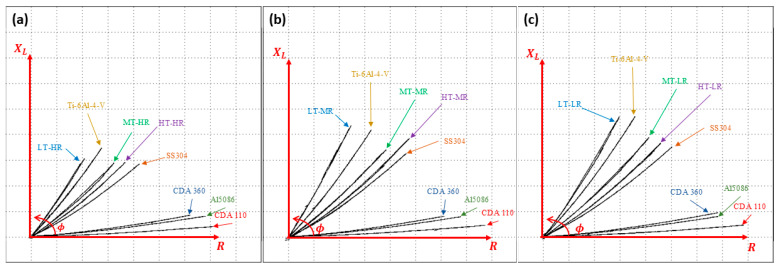
Impedance plots for the Ti-6Al-4V ELI—Nanographene system at 50 KHz. (**a**) High-rugosity samples. (**b**) Medium-rugosity samples. (**c**) Low-rugosity samples. The R represents real part of impedance, the X_L_ denotes imaginary part of impedance, and Φ denotes the phase angle between the real and imaginary components of impedance.

As mentioned in Section 2.5, characterization was performed using the non-destructive eddy current technique. Measurements were taken at different inspection frequencies on all samples. However, no significant results were obtained at high frequencies (i.e., 1 MHz to 6 MHz), as all samples exhibited similar phase angles. This indicates that there were no differences between samples with the induced current inspection. When using lower frequencies between 20 and 70 kHz, however, better differentiation of these phase angles in the impedance plane was observed for the different samples. The inspection frequency with the highest sensitivity was 50 kHz, at which the distance between the angles of the samples became more evident. Figure 8 shows the results obtained using this technique when inspecting parts with the same surface finish and varying substrate thicknesses, as well as integrating reference materials with known conductivity.

Changes in the phase angle of non-ferromagnetic materials have been attributed to changes in electrical conductivity and variations in sample thickness. If the sample has high conductivity, eddy currents tend to concentrate on the surface of the material. This results in less penetration and changes in the phase angle [74]. Conversely, the thickness of the material evaluated using this eddy current technique has been found to make the phase angle more sensitive to thickness when it is less than the penetration depth. This is because, in this case, the currents interact with the bottom of the material.

The procedure used to obtain σ from the phase angle in ECT is consistent with theory and recent models that directly link the phase of coil impedance to the conductivity of non-ferromagnetic materials, thus avoiding complex inversions. This explains why trends in σ reflect trends in the phase angle. Additionally, the 50 kHz frequency maximizes sensitivity in the low/medium conductivity range and millimeter thicknesses, as demonstrated by models and frequency selection criteria for σ measurement by ECT [75].

**Figure 9:**
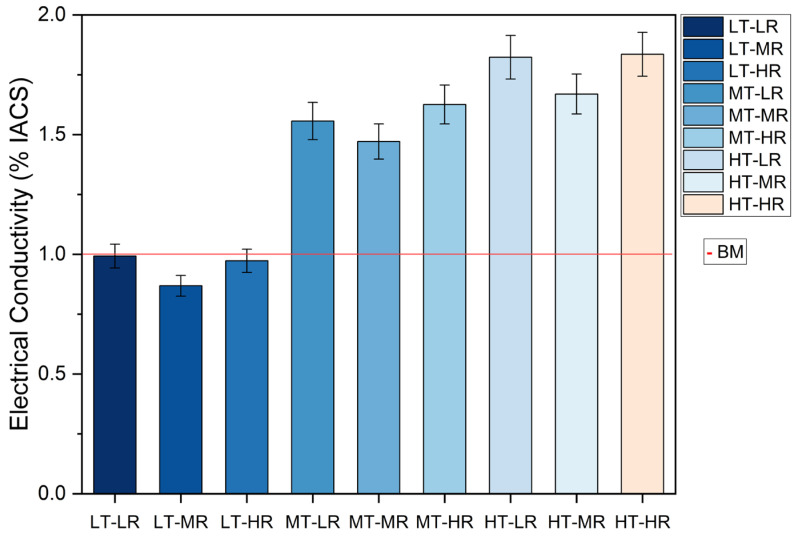
Comparison of electrical conductivity values for Ti-6Al-4V ELI—Nanographene samples and base material.

Finally, conductivity calculations were performed for this technique as described in Section 2.5. Figure 9 shows a graph of the values for the Ti-6Al-4V ELI-Nanographene test specimens, as well as for the base material (BM).

Since these conductivity values were obtained from the phase angle of the samples, it is only natural that they exhibit the same behavior with respect to the thickness of the substrate and its surface roughness. There is a visible difference between pieces of different thicknesses, while the roughness exhibits change without a fixed trend.

### 3.5. Surface Roughness Measurements

Surface roughness values ranging from 0.02 to 0.15 were obtained for the uncoated samples. In contrast, the deposition of nanographene on the Ti-6Al-4V ELI alloy surface resulted in roughness values from 0.07 to approximately 0.23. The precise numerical data for each sample can be found in Table S2 (Supplementary Materials). These results show that values obtained under similar conditions (i.e., with similar surface finishes but different substrate thicknesses) are close with slight variations. These variations can be attributed to the manufacturing process of the different volumes from which the parts were cut, as indicated by Safdar et al. [52] in their investigation of the influence of processing parameters on the surface roughness of Ti-6Al-4V.

### 3.6. Spectroscopy Characterization

**Figure 10:**
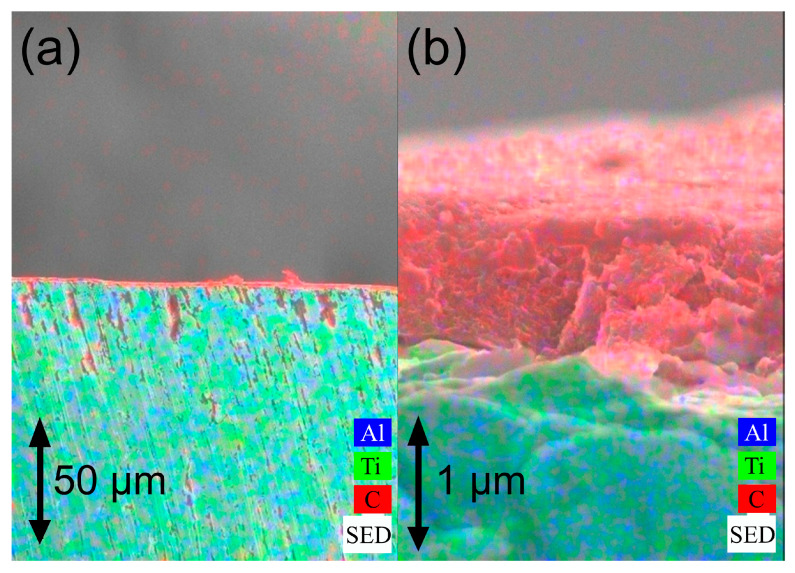
SEM image of (**a**) nanographene coating on Ti-6Al-4V ELI substrate; (**b**) close-up image of nanographene coating on Ti-6Al-4V ELI substrate.

Characterization by scanning electron microscopy is indispensable when dealing with nanographene. The ELI grade Ti-6Al-4V pieces coated with this material were analyzed with this technique, which initially corroborated the existence of a uniform layer on the substrate surface. Figure 10a provides a visual representation of the uniformity of the layer over a substantial portion of the base material. Figure 10b provides a close-up view of this coating. It is also possible to estimate the average thickness of the deposited nanographene layer, which is ~1 micron thickness. The average thickness values for the rest of the samples with different substrate roughness exhibited values between 0.9 and 1.9 μm (see Table S3 of Supplementary Materials for exact values for each sample).

The preceding figure displays a flake-like morphology, which is indicative of nanographene obtained by PECVD technique [76,77].

## 4. Discussion

### 4.1. Plasma-Enhanced Nanographene Growth from Methane on Substrates

Nanographene depositions on diverse substrate materials have been extensively examined across the literature. Among various deposition techniques, PECVD employing methane as a carbon precursor has garnered particular interest due to its low-temperature operation and fine thickness control [78,79,80]. In the PECVD process, copper (Cu) substrates dominate the literature, primarily because Cu’s extremely low carbon solubility [81] at growth temperatures leads to self-restrictive monolayer formation, while its high thermal and electrical conductivity and relative affordability facilitate scalability and nanographene growth. Based on the DFT mechanism, methane undergoes dissociative chemisorption on Cu surfaces with weak Cu–C interactions [82] and low solubility compared to other transitional metals. While other transitional metals (Ni and Co) dissolve carbon into their bulk lattice, leading to multilayer precipitation upon cooling, precious metals (Au, Pd, and Pt) either form carbides or exhibit insufficient catalytic activity by adsorbing carbon onto sub-surface sites, which further prevents uniform/homogenous graphene growth [82].

### 4.2. Proposed Nanographene Pathway Growth

Typical CVD growth mechanisms reported in the literature follow a stepwise pathway [83,84,85,86]: (1) dissociation of methane on the surface; (2) adsorption, surface diffusion, and mobility of precursors on the metal substrate; (3) segregation and nucleation of carbon on the metal substrate; and (4) formation of carbon islands with simultaneous desorption of hydrogen as H_2_ gas. It is important to note that graphene growth is selective on metal substrates; copper (Cu) is widely cited for its low carbon solubility, specifically 1.4 wt-ppm [87], which favors the selective formation of two-dimensional (2D) hexagonal carbon. In this study, we employed PECVD on Ti-6Al-4V, an alloy not commonly used for graphene growth. The solubility of carbon on titanium metal is around 800 wt-ppm at room temperature, but the solubility increases at elevated temperature (600–920 °C) from 2000 to 5000 wt-ppm [88]. However, under optimized conditions, we could observe nanographene growth. To elucidate the growth mechanism, we divided the plausible pathways into three scales, macro, molecular, and atomic, for better understanding.

(1) Activity on Macro-scale: In this study, three polishes (sandpaper meshes 240 and 600) produce different rough surfaces. Increasing the roughness also increases the surface energy and surface area, which further provides an advantage for dissociated precursors to stick/adsorb on substrate.

(2) Molecular scale: Adsorption, diffusion, and chemical affinity: plasma-induced methane dissociation. Suspended above this textured alloy is a low-pressure RF plasma fed by methane and hydrogen. Energetic electrons collide with CH_4_, fragmenting it in a sequence of endothermic steps (ΔH°); the enthalpy gradient refers to the sequence of energy changes (ΔH°) associated with consecutive bond dissociation steps (Equations (3)–(7)) in this chemical reaction as it represents how much energy is required for each step:

CH_4_ → CH_3_* + H* (ΔH° = 4.55 eV) (3)

CH_3_ → CH_2_* + H* (ΔH° = 4.69 eV) (4)

CH_2_ → CH* + H* (ΔH° = 4.76 eV) (5)

CH → C* + H* (ΔH° = 5.76 eV)(6)

H_2_ → H* + H* (ΔH° = 4.50 eV) (7)

Each radical, methyl through atomic carbon, plus atomic hydrogen, is generated in the gas phase (please see Figure S1). Without this energetic dissociation, Ti-6Al-4V at 850 °C would simply accumulate amorphous carbon (coking) because the alloy’s higher carbon affinity draws C atoms beneath the surface rather than allowing them to organize into sp^2^ layers. Once created, CH_X_*and H* species adsorb upon the alloy. Adsorbed CH_X_* fragments diffuse across terraces and step edges, driven by surface energy gradients that correlate with the macro-textured roughness.

**Figure 11:**
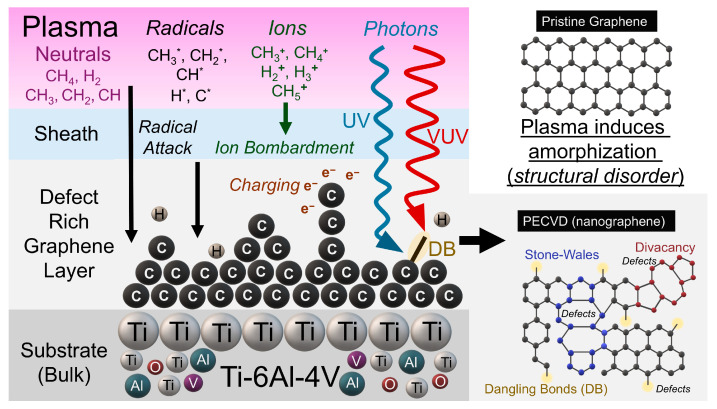
Plausible mechanism pathway, inspired by [93].

(3) Atomic scale: Nucleation, sp^2^ island formation: When local carbon coverage on the plasma-activated Ti-6Al-4V surface surpasses the critical threshold, six-membered sp^2^ rings nucleate preferentially at high-energy sites (defects), deliberately roughened macro-textured surfaces. Atomic hydrogen then selectively etches and stabilizes the process: H* radicals terminate the exposed bonds of nascent sp^3^ clusters, halting 3D coking, while simultaneously removing weakly bound carbon atoms from defective sites, leaving only the planar sp^2^ nuclei intact (see Figure 11). As these hexagonal islands grow laterally, their edges merge into a two-dimensional lattice; simultaneously, H* recombines to form H_2_, desorbing and continually cleaning the surface for further carbon incorporation. The optimized PECVD conditions, with equimolar ratio of methane and hydrogen, yield a continuous nanographene film, as confirmed by Raman spectroscopy: A sharp D band at 1350 cm^−1^ (low I_D_/I_G_) reveals residual sp^3^ defects, a lower G band at 1580 cm^−1^ indicates the in-plane stretching of sp^2^ bonds, and a subtle 2D band “hump” at 2700 cm^−1^ and wider FWHM verified a few layers of graphene [55,60]. It is noteworthy to mention that TiO_2_-covered Ti-6Al-4V is a substrate far less common than copper suitable for nanographene growth.

**Figure 12:**
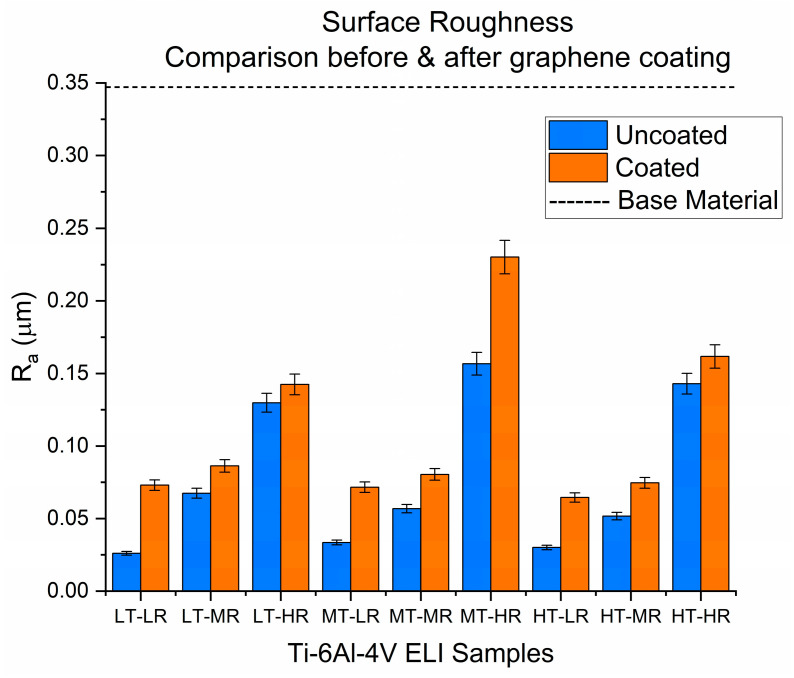
Average surface roughness of Ti-6Al-4V ELI samples.

It is interesting to understand what dictates sp^2^ formation vs. sp^3^ formation during PECVD. The modification from a disordered, 3D “coking” network to a pristine, planar sp^2^ honeycomb centers on a plausible pathway. Firstly, kinetic control arises from the ratio of carbon radical surface diffusion lengths (enhanced at 850 °C) to nucleation sites [73], wherein long diffusion paths [89] and moderate flux steer incoming CH_x_* toward existing islands [90] rather than depositing new, defect-rich clusters. Secondly, hydrogen-assisted selective etching [91,92], where H* preferentially removes sp^3^ and defect sites via a 4.5 eV barrier, simultaneously “cleans” the growing film, ensuring that only the strongest, edge-terminated sp^2^ structures undergo transformation into an atomically perfect lattice. In PECVD, plasma is a complex assortment of electrons, neutrals, radicals, ions, energetic atomic species, and photons (UV/VUV), all of which can certainly promote the deposition of amorphous carbon [93]. Synthesized graphene film that exhibits a strong D band, therefore, indicates a high defect density and an increased fraction of sp^3^ bonding compared with pristine, crystalline graphene. During PECVD, the plasma-sheath region is particularly important where various activities such as radical attack, ion bombardment, and absorption of UV/VUV photons can dissociate weak bonds and generate dangling bonds. Reactive radicals may abstract atoms to produce dangling bonds from sp^3^ sites, so physio-chemical damage often act together. Nunomura (2023) have reviewed plasma-induced defects in the context of semiconductor manufacturing [93,94], and similar defect patterns are observed across a range of plasma chemistries (see Figure 12). Compared with the symmetric hexagonal (6-6-6-6) honeycomb of pristine graphene, these plasma-induced defects disrupt the lattice in several characteristic ways: Bond rotations can convert four hexagons into a 5-7-7-5 Stone–Wales defect [95,96]; removal of one or more carbon atoms produces vacancies that may reconstruct (for example, into a 5-8-5 divacancy) [95,97]; and bond cleavage creates undercoordinated carbon atoms (dangling bonds) that frequently accompany vacancies [93,98]. Collectively, these defects modify the local strain and electronic structure of the material, observed with Raman spectroscopy in this work for PECVD-grown films (see Figure 11).

### 4.3. Thermoelectric Power (TEP) Characterization of Nanographene-Coated Ti-6Al-4V ELI Grade Samples

Obtained values for all electrode systems of TEP tests align with literature-reported Seebeck coefficients for metallic systems (1–10 µV/K, per Tritt [99]) and confirm that the nanographene coating contributes to this thermoelectric response. Notably, these measurements represent one of the first reports of absolute Seebeck coefficient values for nanographene-coated Ti-6Al-4V ELI alloys.

The combined Raman and thermoelectric results indicate that the nanographene coating contributes to the thermoelectric response mainly through interfacial and surface effects. The defect density inferred from Raman spectroscopy increases carrier scattering within the coating, explaining why the Seebeck coefficient remains dominated by the metallic substrate while still exhibiting sensitivity to surface morphology and electrode configuration.

In addition, the consistent trends observed across all samples—regardless of thickness or surface finish—reinforce the robustness of the TEP technique for non-destructive assessment. Samples with mirror-polished finishes systematically exhibited higher Seebeck coefficients than their rougher counterparts, suggesting surface texture influences the thermoelectric response of the nanographene layer. Moreover, to the best of our knowledge, there are no prior reports of absolute Seebeck coefficient values obtained via TEP measurements for nanographene-coated Ti-6Al-4V ELI systems. Although Amollo et al. [100] reported Seebeck values around 10 µV/K for monolayer graphene synthesized on Si/SiO_2_/Ni substrates, their findings pertain to a different substrate–coating combination. The values obtained in the present study (1.9–3.4 µV/K) fall within the typical range for metals and may reflect the combined effect of the metallic substrate and nanographene architecture.

Differences in work function, chemical stability, and contact resistance at the metal–nanographene interface play a key role in the observed thermoelectric sensitivity. The Au–Cu system minimizes parasitic interfacial effects, allowing the thermoelectric signal to more faithfully reflect surface-related variations rather than electrode-induced artifacts.

### 4.4. Eddy Currents Testing (ECT) Conductivity Measurements of Nanographene-Coated Ti-6Al-4V ELI Grade Samples

At 50 kHz, the penetration depth (δ) of Ti-6Al-4V is approximately millimeters. With substrates measuring 1.6, 3.2, and 7 mm, the induced field is largely influenced by the substrate’s volume rather than the nanographene surface layer’s thickness, which is approximately 1–2 µm. As the thickness increases, therefore, the phase angle decreases and the deduced apparent conductivity increases, as expected when the thickness exceeds or approaches δ.

However, although nanographene is very thin with respect to δ (its direct electromagnetic contribution is small), there are interface and stress state mechanisms that can slightly alter the apparent conductivity compared to the bare material.

Titanium is a strong carbide former. At high CVD/PECVD temperatures (approximately 800–900 °C), interfacial chemistry can favor TiC or local Ti–C phases. This creates more conductive electrical pathways near the surface that partially “see” the induced currents. This is equivalent to a very thin, more conductive layer in parallel with the substrate. The literature reports the propensity to form TiC at Ti–graphene/carbon interfaces and the difficulty of growing graphene on carbide-forming metals due to this competitive reaction. These scenarios support a small increase in apparent σ after coating [101,102].

On the other hand, roughness modifies the local distribution of currents and can skew σ measurements by ECT by up to ~10–20% in severe cases, such as shot peening. On smoother surfaces, like those used here, the effect is smaller, though not zero, and it depends on frequency. This explains the small differences with no clear trend between LR, MR, and HR, which are superimposed on the dominant effect of thickness [103].

Because the penetration depth at 50 kHz exceeds the nanographene thickness by approximately three orders of magnitude, the measured phase angle and apparent conductivity primarily reflect substrate-controlled eddy-current behavior. However, the presence of nanographene and interfacial Ti–C phases modifies the near-surface electrical response sufficiently to produce measurable and reproducible shifts, validating the choice of 50 kHz as the optimal inspection frequency for this system.

Additionally, various studies demonstrate that graphene coatings or graphene-based composites on titanium or alloys can enhance the surface’s or functional coatings’ effective electrical conductivity (even when the substrate dominates) by improving electronic percolation and reducing local contact resistances [104,105]. This is consistent with the slight shift in σ observed compared to the uncoated sample.

### 4.5. Surface Roughness Measurements

As for the roughness values, the trend observed in Figure 12 is noteworthy. In general, the roughness value increases when the coating is deposited on the surface of the sample. This coincides with the findings of Mohamed and Nabey [106], who deposited Co-graphene films on a steel substrate and found that the graphene in the coating films increased the surface roughness of the system. This is attributed to the graphene forming a thin, porous network that causes the material to exhibit intrinsic corrugation. This phenomenon has also been studied and reported by Geringer et al. [107]. However, as can be seen, although the roughness values increase in the presence of the graphene coating, they remain in a similar order of magnitude to those of the material without this surface modification. This is mainly because graphene layers typically conform to the surface’s contours, i.e., they follow the substrate’s texture, as indicated by the research of Lui et al. [108] and Stöberl et al. [77].

Beyond delineating alterations in Ra, the impact of surface roughness on the measured Seebeck coefficient can be elucidated through the framework of surface and interface scattering concepts. The presence of increased roughness leads to an augmented density of asperities and local height gradients. This, in turn, results in enhanced electron scattering within the near-surface region, consequently reducing the effective carrier mean free path, thereby decreasing the thermoelectric response detected at the surface. This interpretation aligns with classical thin-film transport frameworks, wherein surface roughness and grain-boundary scattering mechanisms contribute to the modification of charge transport (e.g., Fuchs–Sondheimer-type surface scattering and its associated extensions). Furthermore, the presence of roughness has been shown to influence the actual contact area and the stability of the metal–nanographene junction during hot-tip measurements. This interaction has the capacity to amplify or attenuate the measured thermoelectric signal, contingent upon the electrode material utilized. This mechanistic interpretation is further substantiated by the statistical results reported in the Supplementary Materials, wherein surface roughness exhibits a robust negative correlation with the Au–Cu thermoelectric response. This observation signifies that morphology-driven scattering and interfacial coupling emerge as the predominant secondary factors that modulate Seebeck measurements within this system.

### 4.6. SEM Characterization

The morphology observed in Figure 10, in the close-up of the nanographene coating on the Ti-6Al-4V ELI substrate, is also visible in the other SEM images. This demonstrates that the nanographene deposits are consistent, regardless of the roughness of the Ti-6Al-4V ELI substrate (see Figures S2–S10 in the Supplementary Materials: SEM images for all samples).

The formation of these flake-like structures is attributable to the elevated temperatures at which nanographene deposition is undertaken using the PECVD technique (in this study, temperatures analogous to those used in conventional CVD are achieved). Consequently, once these temperatures are attained, dendritic structures are formed, and upon entering the cooling stage, these structures begin to take an irregular shape with edges that end in the form of “flakes”. These flakes are the product of tensions caused by the difference in thermal coefficients between the nanographene and the substrate [109,110].

### 4.7. Functional and Statistical Basis for Potential Applications

The observed variations in Seebeck coefficient values and surface roughness across sample groups, as statistically analyzed through two-factor ANOVA and a correlation matrix (see Supplementary Materials, Tables S4–S6), provide a functional basis for potential biomedical applications. Specifically, the negative correlation between roughness and thermoelectric response supports the possibility of optimizing surface morphology to enhance energy harvesting performance in implantable or wearable devices. The thermoelectric properties (Seebeck 1.9–3.4 µV/K) and tunable surface roughness demonstrated by our nanographene-coated Ti-6Al-4V system open avenues for integration into smart biomedical interfaces and wearable/implantable thermal sensors. Graphene-based electrode materials have already shown enhanced interfacing with neural tissue and high electrochemical stability [38,111,112], while wearable thermoelectric generators using graphene/conductive polymer (PEDOT: PSS) have achieved Seebeck values in the 25–150 µV/K range [113]. Furthermore, porous graphene-foam sensors have been used to detect both temperature (resolution ~0.5 °C) and strain via thermoelectric mechanisms [112]. These examples support the potential of our coating to serve as a multifunctional biocompatible interface that harvests thermal energy and senses physiological changes—particularly when optimized for surface morphology and electrical/thermal coupling.

## 5. Conclusions

In light of the preceding discussion, we conclude the feasibility of depositing nanographene films on Ti and Ti-6Al-4V alloy ELI grade by PECVD at temperatures of 850 °C with an equimolar mixture of CH_4_/H_2_ gases. This process is conducted under vacuum pressure and requires 15 min of plasma exposure.

The nanographene coatings obtained through PECVD with the aforementioned parameters are uniform.

Of the three tip systems with which the samples were evaluated by thermoelectric potential, the Au-Cu pair demonstrated heightened sensitivity to variations in the Seebeck coefficient induced by disparate factors.

The 50 kHz frequency facilitated enhanced sensitivity in differentiating samples using the phase angle, in contrast to high frequencies (1–6 MHz), where the distinctions between samples were negligible. Substrate thickness exhibited a substantial influence on the alterations in phase angle and electrical conductivity for the samples examined. In addition to demonstrating an inversely proportional relationship with angle and a directly proportional relationship with conductivity, it was observed that the penetration depth of the induced currents differed significantly.

The presence of nanographene films on the surface of ELI grade Ti-6Al-4V can be corroborated by SEM and AFM, and the coating uniformity and thickness were determined with greater precision.

The nanographene-coated Ti-6Al-4V ELI alloy developed in this study exhibits distinct advantages in terms of surface passivation, micrometric thickness control, and tunable roughness, all of which contribute to enhanced physicochemical stability. Notably, the coatings demonstrated measurable thermoelectric activity, with Seebeck coefficients ranging from 1.9 to 3.4 µV/K, and surface roughness values (Ra) spanning from 0.08 to 0.42 µm, depending on the substrate preparation. These features are particularly attractive for the development of multifunctional biomedical platforms, where controlled topography enhances biocompatibility and thermoelectric performance enables passive sensing or energy harvesting. Such properties position this system as a promising candidate for integration into smart biomedical interfaces, wearable or implantable temperature sensors, and thermoelectric generators (TEGs) designed for physiological environments.

Notably, the nanographene coating produces little change in surface roughness, which indicates strong adhesion to the Ti-6Al-4V substrate; this good interfacial contact enhances heat transfer across the coating–substrate boundary and, therefore, improves the sensitivity and reliability of thermal detection in wearable devices. While the present results demonstrate initial coating characterization and performance, further work is needed to assess long-term stability in bodily fluids, thermoelectric behavior under repeated thermal cycling, and interface performance in biological environments. Recent studies have demonstrated the potential of nanographene-based materials in flexible TEGs and porous sensor foams capable of converting small thermal gradients into electrical signals, supporting their utility in autonomous health-monitoring devices. By tailoring both morphology and electrical response, the present system aligns with the requirements for robust, biocompatible, and energy-responsive surfaces suitable for next-generation medical applications.

## Data Availability

The original contributions presented in this study are included in the article/Supplementary Material. Further inquiries can be directed to the corresponding author.

## Supplementary Materials

The following supporting information can be downloaded at: https://www.mdpi.com/article/10.3390/ma19040774/s1, Table S1: I_2D_/I_G_ and I_D_/I_G_ Ratios for Graphene Coatings on Ti substrates obtained from Raman spectra; Table S2: Average Roughness values for Graphene-coated and uncoated Ti-6Al-4V ELI samples; Table S3: Average Thickness Values for Graphene Coatings on Ti-6Al-4V ELI substrates; Table S4: Two-way ANOVA results for coating thickness; Table S5: Two-way ANOVA results for surface roughness; Table S6: Correlation matrix of measured parameters. Figure S1: Methane plasma OES; Figure S2: SEM image of graphene-coated sample (LT-LR); Figure S3: SEM image of graphene-coated sample (LT-MR); Figure S4: SEM image of graphene-coated sample (LT-HR); Figure S5: SEM image of graphene-coated sample (MT-LR); Figure S6: SEM image of graphene-coated sample (MT-MR); Figure S7: SEM image of graphene-coated sample (MT-HR); Figure S8: SEM image of graphene-coated sample (HT-LR); Figure S9: SEM image of graphene-coated sample (HT-MR); Figure S10: SEM image of graphene-coated sample (HT-HR).

## Abbreviations

The following abbreviations are used in this manuscript:

CVD	Chemical Vapor Deposition
PECVD	Plasma-Enhanced Chemical Vapor Deposition
NG	Nanographene
TEP	Thermoelectric Power
SEM	Scanning Electron Microscopy
LT	Low Thickness (1.6 mm samples)
MT	Medium Thickness (3.2 mm samples)
HT	High Thickness (7 mm samples)
LR	Low Roughness (samples mirror polished)
MR	Medium Roughness (samples prepared with 600 microns sandpaper)
HR	High Roughness (samples prepared with 240 microns sandpaper)
