# Supplementary material for: Plasma-Enhanced Graphene Coatings on Ti-6Al-4V: Insights from Non-Destructive Characterization
Source: Materials (Basel). 2026 Feb 16;19(4):774. doi: 10.3390/ma19040774 (PMC12941520; doi:10.3390/ma19040774)
Supplement: Supplementary file 1 [file materials-19-00774-s001.zip › materials-4143375-supplementary.pdf]

# Plasma-Enhanced Graphene Coatings on Ti-6Al-4V: Insights from Non-Destructive Characterization

**Victor J. Sagrero [1], Fnu Gorky [2], Vashanti Storr [2], Fernando M. de Oliveira [3], Héctor G. Carreón [1] and María L. Carreón [2,*]**

[1] Instituto de Investigación en Metalurgia y Materiales, Universidad Michoacana de San Nicolás de Hidalgo, Morelia, Michoacán 58030, Mexico; victor.sagrero@umich.mx (V.J.S.); hcarreon@umich.mx (H.G.C.)
[2] Ralph E. Martin Department of Chemical Engineering, University of Arkansas, Fayetteville, AR 72701, USA; gorky@uark.edu (F.G.); vstorr@uark.edu (V.S.)
[3] Institute for Nanoscience and Engineering, University of Arkansas, Fayetteville, AR 72701, USA; fmaiade@uark.edu
* Correspondence: mc138@uark.edu

## 1. $I_D/I_G$ & $I_{2D}/I_G$ ratio table

Five measurements were performed by Raman spectroscopy to obtain the intensity of the different bands in the sample that exhibited the characteristic graphene peaks with the optimal parameters. The ratios of intensities of these bands are shown to be associated with the number of deposited graphene layers (thickness) and the concentration of defects in the coating of this material.

Table S1. $I_{2D}/I_G$ and $I_D/I_G$ ratios for graphene coatings on Ti substrates obtained from Raman spectra.

| Measure | D Band | G Band | 2D Band | I2D/IG Ratio (thickness) | ID/IG Ratio (defects) |
|---|---|---|---|---|---|
| **1** | 1615.5211 | 1364.7013 | 769.32355 | 0.563 | 1.183 |
| **2** | 1612.1711 | 1306.3013 | 766.0285 | 0.586 | 1.234 |
| **3** | 2584.2026 | 1950.0101 | 855 | 0.438 | 1.325 |
| **4** | 2307.6519 | 1825.4167 | 845.33704 | 0.463 | 1.264 |
| **5** | 1633.7543 | 1332.3602 | 817.55341 | 0.613 | 1.226 |

## 2. Surface Roughness values for all samples

Table S2 shows all the average surface roughness values obtained for the different Ti-6Al-4V ELI samples with and without graphene coating, by the measurements performed with the Mitutoyo SJ-210 roughness tester.

Table S2. Average roughness values for graphene-coated and uncoated Ti-6Al-4V ELI samples.

| Sample ID | $R_a$ Uncoated (μm) | $R_a$ Coated (μm) |
|---|---|---|
| LT-LR | 0.026 | 0.073 |
| LT-MR | 0.06745 | 0.0863 |

| | | |
|---|---|---|
| LT-HR | 0.1298 | 0.1424 |
| MT-LR | 0.0335 | 0.0716 |
| MT-MR | 0.0568 | 0.0804 |
| MT-HR | 0.1567 | 0.2302 |
| HT-LR | 0.03005 | 0.0645 |
| HT-MR | 0.0517 | 0.0746 |
| HT-HR | 0.1429 | 0.1617 |

## 3. Coating thickness values

Table S3 summarizes the average thickness values obtained for each of the samples analyzed using SEM and SigmaScan Pro image processing.

Table S3. Average thickness values forgGraphene coatings on Ti-6Al-4V ELI substrates.

| Sample | Average Coating Thickness (μm) | St. Deviation | St. Error | Max. 95% Confidence interval | Min. 95% Confidence interval |
|---|---|---|---|---|---|
| LT-LR | 1.057 | 0.037 | 0.012 | 1.069 | 1.045 |
| LT-MR | 0.916 | 0.025 | 0.008 | 0.924 | 0.909 |
| LT-HR | 1.094 | 0.034 | 0.011 | 1.105 | 1.083 |
| MT-LR | 1.141 | 0.100 | 0.031 | 1.173 | 1.110 |
| MT-MR | 1.531 | 0.148 | 0.047 | 1.578 | 1.484 |
| MT-HR | 1.360 | 0.111 | 0.035 | 1.395 | 1.325 |
| HT-LR | 1.905 | 0.095 | 0.030 | 1.935 | 1.875 |
| HT-MR | 1.735 | 0.084 | 0.027 | 1.761 | 1.708 |
| HT-HR | 1.519 | 0.230 | 0.073 | 1.592 | 1.447 |

## 4. Methane plasma optical emission spectra

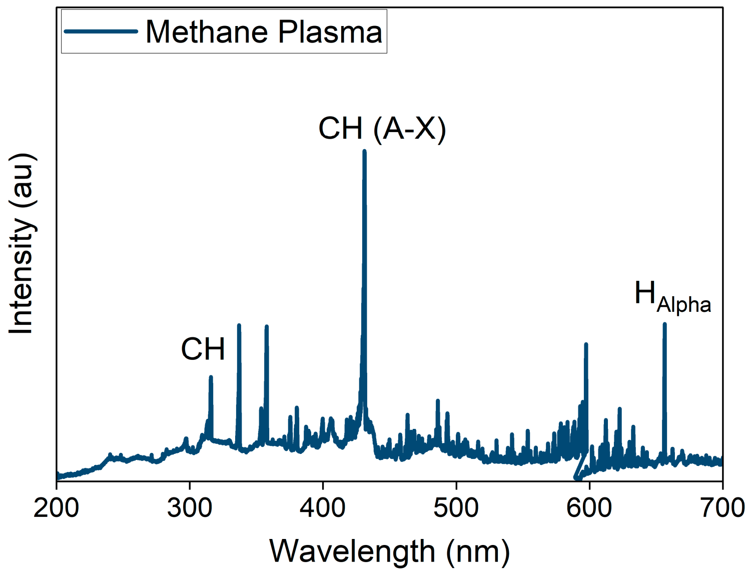


Figure S1: Methane plasma OES.

## 5. SEM images of graphene-coated Ti-6Al-4V ELI samples

Representative SEM micrographs are shown below for each combination of substrate thickness and surface finish, where the low and medium-thickness set of samples use green for the Ti substrate and red for the graphene coating, while the high-thickness set has these colors inverted:

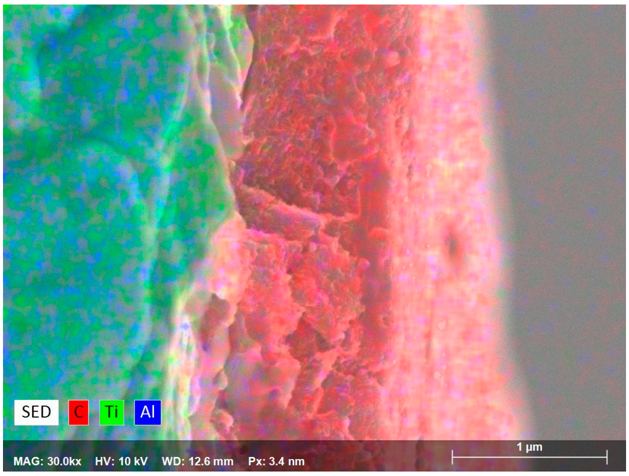


Figure S2: SEM image of graphene-coated sample (LT-LR).

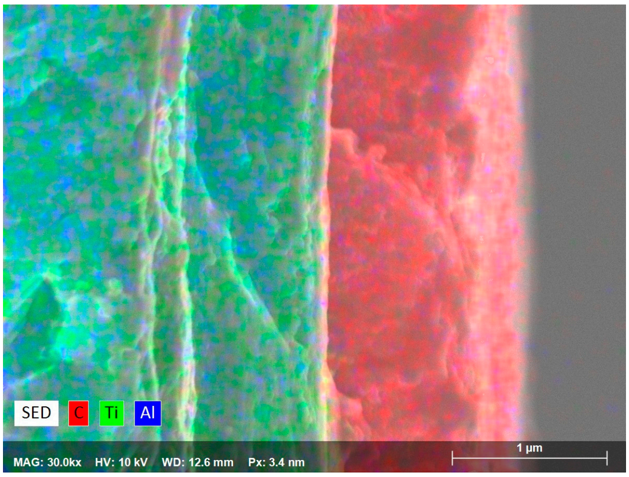


Figure S3: SEM image of graphene-coated sample (LT-MR).

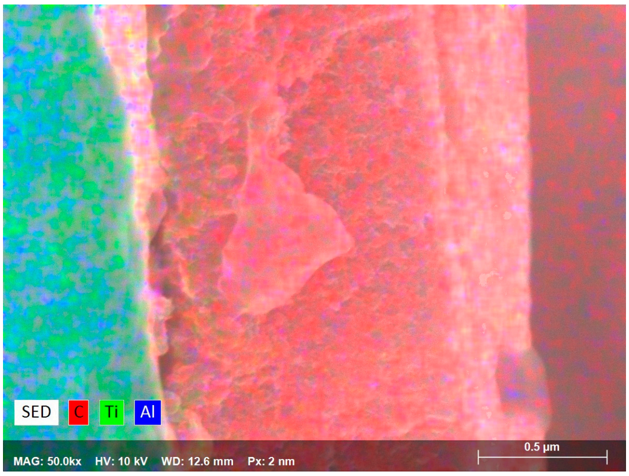


Figure S4: SEM image of graphene-coated sample (LT-HR).

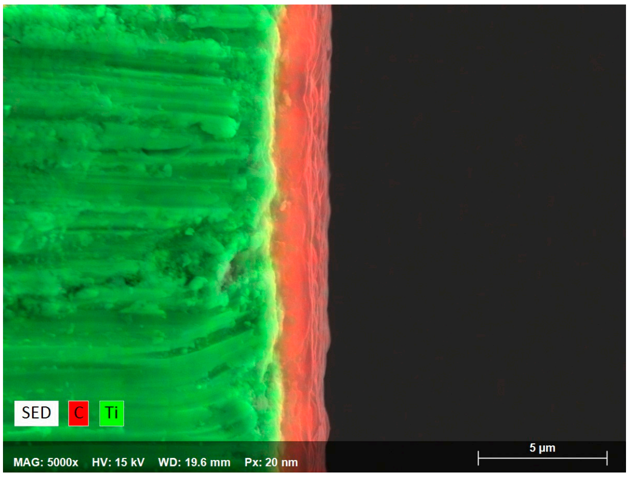


Figure S5: SEM image of graphene-coated sample (MT-LR).

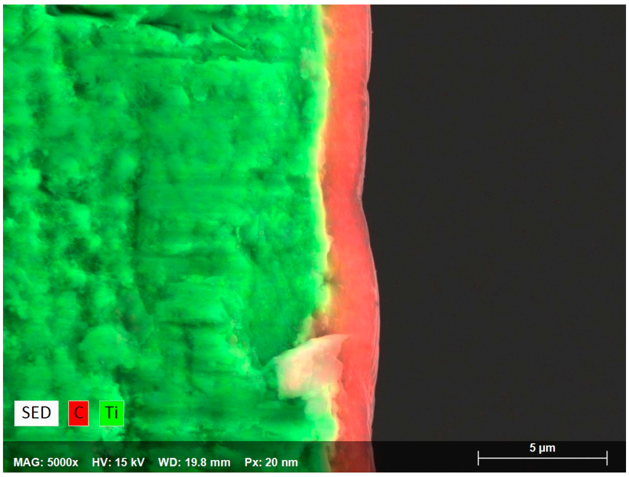


Figure S6: SEM image of graphene-coated sample (MT-MR).

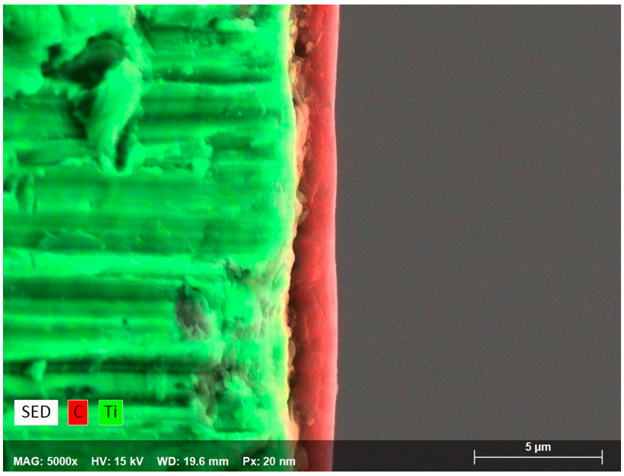


Figure S7: SEM image of graphene-coated sample (MT-HR).

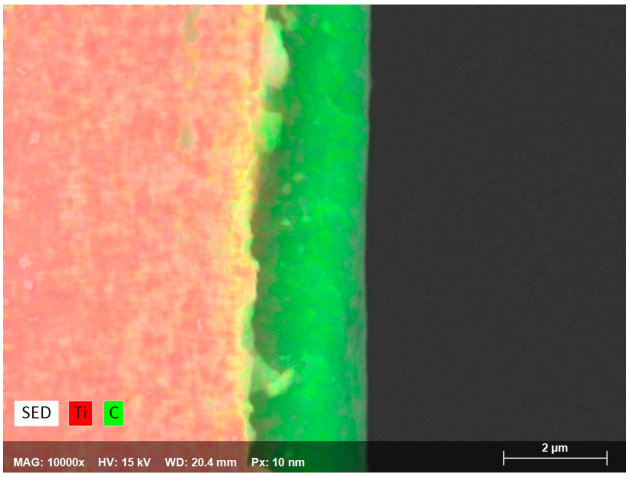


Figure S8: SEM image of graphene-coated sample (HT-LR).

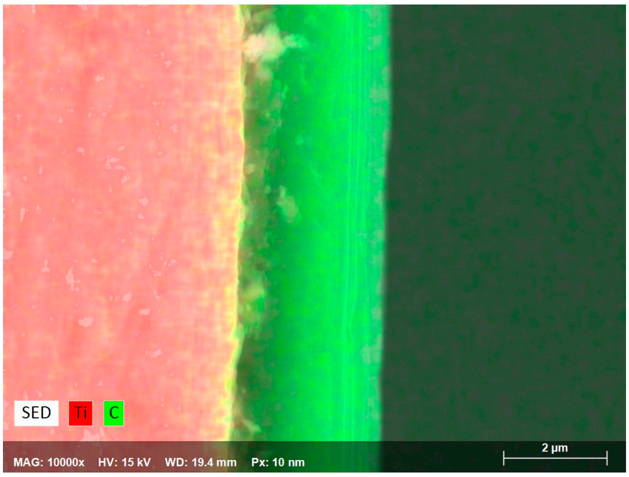


Figure S9: SEM image of graphene-coated sample (HT-MR).

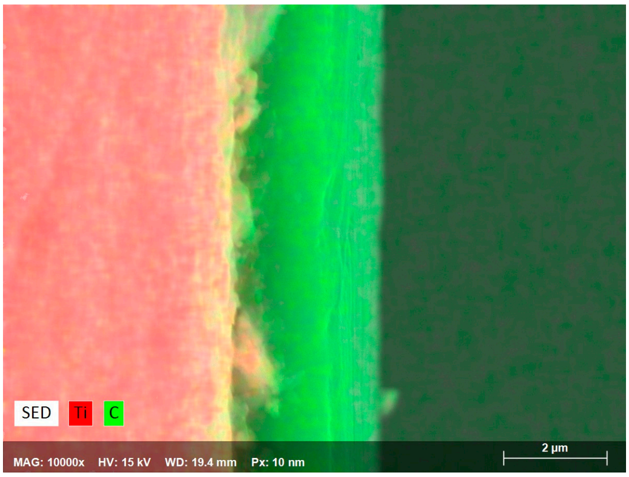


Figure S10: SEM image of graphene-coated sample (HT-HR).

## 6. Statistical Analysis and Supplementary Tables

This section presents the statistical treatment of data related to the graphene coating thickness, surface roughness and TEP measurements, with the latter being incorporated exclusively within the correlation matrix.

Two-factor ANOVA was applied to evaluate the influence of substrate thickness and surface finish on the following response variables: coating thickness (measured from SEM using SigmaScan Pro) and surface roughness (Ra values from Mitutoyo SJ-210).

The correlation matrix is a tool that can be used to visualize the Pearson correlation coefficient between each of the variables in the matrix. The correlation coefficient ranges from -1 to 1, where

a negative value of unity represents an inverse correlation between the variables, a positive value indicates a direct correlation, and values close to 0 indicate the absence of such correlation.

Table S4. Two-way ANOVA results for coating thickness.

| **Thickness / Surface Finish** | **LR** | **MR** | **HR** | | | |
|---|---|---|---|---|---|---|
| LT | 1.0572 | 0.9163 | 1.0943 | | | |
| MT | 1.1412 | 1.5309 | 1.3596 | | | |
| HT | 1.9048 | 1.7349 | 1.5192 | | | |
| | | | | | | |
| Anova: Two-Factor Without Replication | | | Rows = Substrate Thickness | Columns = Surface Finish | | |
| | | | | | | |
| *SUMMARY* | *Count* | *Sum* | *Average* | *Variance* | | |
| LT | 3 | 3.0678 | 1.022591671 | 0.008820577 | | |
| MT | 3 | 4.0317 | 1.343891604 | 0.038143501 | Criterion: | If P-value < 0.05 |
| HT | 3 | 5.1589 | 1.719648089 | 0.037351204 | **The effect is statistically significant** | |
| | | | | | | |
| LR | 3 | 4.1033 | 1.367759717 | 0.218095907 | | |
| MR | 3 | 4.1821 | 1.394019535 | 0.181588776 | | |
| HR | 3 | 3.9731 | 1.324352113 | 0.046074042 | | |
| | | | | | | |
| ANOVA | | | | | | |
| *Source of Variation* | *SS* | *df* | *MS* | *F* | *P-value* | *F crit* |
| Rows | 0.730314234 | 2 | 0.365157117 | 9.060789834 | 0.032695479 | 6.94427191 |
| Columns | 0.007427348 | 2 | 0.003713674 | 0.092148879 | 0.913849796 | 6.94427191 |
| Error | 0.161203217 | 4 | 0.040300804 | | | |
| | | | | | | |
| Total | 0.898944799 | 8 | | | | |

Table S5. Two-way ANOVA results for surface roughness.

| **Thickness / Surface Finish** | **LR** | **MR** | **HR** | | | |
|---|---|---|---|---|---|---|
| LT | 0.073 | 0.0863 | 0.1424 | | | |
| MT | 0.0716 | 0.0804 | 0.2302 | | | |
| HT | 0.0645 | 0.0746 | 0.1617 | | | |
| | | | | | | |
| Anova: Two-Factor Without Replication | | | Rows = Substrate Thickness | Columns = Surface Finish | | |
| | | | | | | |
| *SUMMARY* | *Count* | *Sum* | *Average* | *Variance* | | |
| LT | 3 | 0.3017 | 0.100566667 | 0.001356743 | | |
| MT | 3 | 0.3822 | 0.1274 | 0.00794524 | Criterion: | If P-value < 0.05 |
| HT | 3 | 0.3008 | 0.100266667 | 0.002856043 | **The effect is statistically significant** | |
| | | | | | | |
| LR | 3 | 0.2091 | 0.0697 | 0.00002077 | | |
| MR | 3 | 0.2413 | 0.080433333 | 3.42233E-05 | | |
| HR | 3 | 0.5343 | 0.1781 | 0.00212893 | | |
| | | | | | | |
| ANOVA | | | | | | |
| *Source of Variation* | *SS* | *df* | *MS* | *F* | *P-value* | *F crit* |
| Rows | 0.001456336 | 2 | 0.000728168 | 1.000398419 | 0.444326418 | 6.94427191 |
| Columns | 0.021404542 | 2 | 0.010702271 | 14.70339037 | 0.014336751 | 6.94427191 |
| Error | 0.002911511 | 4 | 0.000727878 | | | |
| | | | | | | |
| Total | 0.025772389 | 8 | | | | |

Table S6. Correlation matrix of measured parameters.

| | *Substrate Thickness (mm)* | *Coating Thickness (µm)* | *Rugosity, Ra (µm)* |
|---|---|---|---|
| Substrate Thickness (mm) | 1 | | |
| Coating Thickness (µm) | 0.885802417 | 1 | |
| Rugosity, Ra (µm) | -0.056654105 | -0.088150885 | 1 |
| TEP Cu ΔT = 20 | -0.626290878 | -0.661179526 | -0.121093543 |
| TEP Cu ΔT = 30 | 0.02688377 | -0.097029598 | -0.77874928 |
| TEP Cu ΔT = 40 | 0.24155389 | 0.079703188 | -0.646841543 |
| TEP Cu ΔT = 50 | 0.232122833 | 0.281971904 | 0.067212499 |
| TEP Ni ΔT = 20 | 0.644663796 | 0.528083932 | -0.705305951 |
| TEP Ni ΔT = 30 | 0.297825114 | 0.194520705 | -0.672233511 |
| TEP Ni ΔT = 40 | 0.61660933 | 0.639614459 | -0.569543041 |
| TEP Ni ΔT = 50 | 0.335618257 | 0.365337135 | -0.799767947 |
| TEP Au ΔT = 20 | -0.138834566 | -0.029752397 | -0.048871549 |
| TEP Au ΔT = 30 | 0.085835921 | -0.0442537 | -0.880054767 |
| TEP Au ΔT = 40 | 0.039070619 | -0.069142756 | -0.762741054 |
| TEP Au ΔT = 50 | 0.107024578 | 0.110318152 | -0.904118181 |

The columns to the right of the roughness are omitted because they only indicate the interaction between gradients and spike systems for the thermoelectric power, which is not relevant for the present study.

A heat map is also presented, in which the intense green coloration denotes a Pearson coefficient approximating 1, and the intense red signifies values approaching -1. The findings of this study demonstrate a substantial statistical correlation between substrate thickness and coating thickness. However, neither of these parameters exhibited a statistically significant relationship with the surface roughness of the samples or the thermoelectric potential values obtained. Conversely, the roughness exhibited a high negative correlation with various PTE systems, with the most significant correlations observed for gold tips with gradients of 30 and 50 degrees. This suggests a potential relationship between the material's surface finish and the values obtained by the non-destructive evaluation technique of thermoelectric power.
